# Identification of CTSC-driven progression in ESCC by single-cell sequencing and experimental validation

**DOI:** 10.3389/fimmu.2025.1585139

**Published:** 2025-07-16

**Authors:** Xin Sui, Yongxu Jia, Jing Li, Jiayao Xu, Wenjia Wang, Yanru Qin

**Affiliations:** Department of Clinical Oncology, The First Affiliated Hospital Zhengzhou University, Zhengzhou, Henan, China

**Keywords:** esophageal squamous cell carcinoma, tumor microenvironment, single cell RNA sequencing, malignant cell, apoptosis, CTSC

## Abstract

**Background:**

The progression of cancer cells is influenced by the tumor microenvironment (TME); however, the molecular mechanisms driving the progression of esophageal squamous cell carcinoma (ESCC) remain unclear. Therefore, we aimed to investigate the TME of ESCC and construct a risk signature based on apoptosis-related genes to identify prognosis-related genes in ESCC.

**Methods:**

We integrated a total of 92,714 cells from 18 samples across three single-cell datasets to analyze the differences in cellular landscapes between primary tumor tissues and adjacent normal tissues. Subsequently, univariate COX regression analysis was employed to construct an apoptosis-related prognostic risk model. The expression of key risk genes was elucidated using immunohistochemistry (IHC). Additionally, the effects of CTSC knockdown on ESCC cell behavior were validated through *in vitro* and *in vivo* experiments.

**Results:**

We identified three malignant cell subtypes (Malig1, Malig2, and Malig4) associated with worse prognosis, which were enriched in apoptosis-related pathways. Pseudotime analysis revealed that the expression scores of apoptosis-related pathways increased along the inferred pseudotime, indicating that apoptosis plays a critical regulatory role in the differentiation of malignant epithelial cells. Furthermore, analysis of the TME demonstrated that immune cells and cancer-associated fibroblasts (CAFs) were significantly more abundant in tumor tissues compared to non-tumor tissues. Additionally, we identified eight apoptosis-related genes associated with prognosis, among which the expression of CTSC was closely correlated with resistance outcomes in patients receiving neoadjuvant immunotherapy. *In vitro* experiments showed that knockdown of CTSC inhibited the proliferation, migration, and other processes of ESCC cells. *In vitro* experiments showed that knockdown of CTSC inhibited tumor growth and expression of fibroblast markers.

**Conclusions:**

CTSC plays a crucial role in driving TME remodeling and the progression of drug resistance in ESCC, making it a potential target for clinical therapy.

## 1 Introduction

Esophageal cancer (EC) is the eighth leading cause of cancer-related deaths worldwide (1). Approximately 85% of EC cases are classified as squamous cell carcinoma of the esophagus, which accounts for nearly 300,000 deaths annually due to its extreme aggressiveness (2, 3). Currently, the standard treatments for EC include surgery, chemotherapy, radiotherapy, and immunotherapy (4). Unfortunately, despite advancements in treatment strategies for ESCC, some patients experience worse clinical outcomes, with 5-year survival rates of less than 25% (5). In recent years, immune checkpoint blockade therapy has demonstrated promising efficacy in patients with metastatic advanced ESCC (6–8). However, some patients do not respond to immune checkpoint blockade therapy, which may be attributed to the heterogeneity of individual tumor immune cell composition (9). There is currently no effective strategy to analyze the relationship between the diversity of the ESCC microenvironment and the malignant phenotype and drug resistance of ESCC, which impedes the development of precision therapeutic immunotherapies for ESCC patients.

The tumor microenvironment (TME) is a multicellular context characterized by complex interactions between stroma and tumor cells (10). Aberrant tumor proliferation, angiogenesis, inhibition of apoptosis, mechanisms of drug resistance, and evasion of immune surveillance are all intrinsically linked to the TME (11, 12). Previous studies have demonstrated that stromal cells, including T cells, macrophages, and fibroblasts, as well as malignant cells, exhibited significant heterogeneity within the TME of ESCC (13). Furthermore, genomic alterations in both immune and stromal cells may influence their interactions with cancer cells, thereby affecting tumor progression and responses to anticancer therapies (14–16). For instance, Ren et al. identified nine genes that were differentially expressed in cancer-associated fibroblasts (CAFs) in ESCC, which were prognostically significant and could serve as independent prognostic factors for ESCC (17). Additionally, the failure of anti-angiogenic drug therapies was believed to result from metabolic adaptation and reprogramming of cancer cells, as well as abnormalities in endothelial cells and their interactions with pericytes (18).

In recent years, single-cell RNA sequencing (scRNA-seq) technology has revolutionized the study of cancer progression and drug resistance by providing novel insights into complex cellular heterogeneity (19, 20). Analyzing gene expression networks at the single-cell level enables high-resolution characterization of cellular heterogeneity, as well as the development and differentiation status in diverse systems (13). In this study, we explored the cellular landscape of ESCC based on scRNA-seq datasets. The TME remodeling during ESCC malignant progression was comprehensively characterized. Additionally, we identified eight apoptosis-related genes associated with prognosis, among which the expression of CTSC was closely linked to drug resistance outcomes of patients treated with neoadjuvant immunotherapy. Furthermore, CTSC promoted tumor progression in ESCC. Accordingly, CTSC plays a crucial role in driving TME remodeling and the progression of resistance in ESCC, making it a potential target for clinical treatment.

## 2 Materials and methods

### 2.1 scRNA-seq data collection

The GSE196756, GSE188900, GSE221561 and GSE203115 datasets were downloaded from the Gene Expression Omnibus (GEO, https://www.ncbi.nlm.nih/). The GSE196756 dataset comprised 6 samples in total: 3 tumor samples from 3 treatment-naïve ESCC patients and 3 paired adjacent normal samples, all of which were included in our analysis. The GSE188900 dataset included 8 samples: 7 tumor samples from 5 treatment-naïve ESCC patients and 1 distal normal sample, all incorporated into this study. The GSE221561 dataset contained 11 samples: 7 tumor samples from 7 ESCC patients who received neoadjuvant therapy, plus 2 tumor samples and 2 paired adjacent normal samples from 2 ESCC patients undergoing surgery alone. For this study, we specifically selected only the 2 tumor samples and 2 paired adjacent normal samples from the surgery-only patients.

### 2.2 General transcriptome data collection

Transcriptome expression matrices for a total of 102 ESCC patient samples were downloaded from The Cancer Genome Atlas (TCGA, https://portal.gdc.cancer.gov/). Among them, 99 ESCC tumor samples have complete prognostic information.

The GSE75241 dataset was downloaded from the GEO dataset (https://www.ncbi.nlm.nih.gov/geo/) and contains the complete transcriptome sequencing results from 15 ESCC tumor samples and their matched noncancerous mucosal samples.

### 2.3 scRNA-seq data processing and cellular annotation

The single-cell RNA sequencing data were downloaded from GEO database in preprocessed matrix format. These data had already undergone alignment to the human reference genome (GRCh38) and gene expression quantification using Cell Ranger (v6.1.2). We therefore performed downstream analysis directly using these preprocessed expression matrices. The scRNA-seq data were then converted to Seurat objects using the Seurat package (v4.1.1) of the R software. First, Seurat objects were created using the CreateSeuratObject function and the parameter min.cells was set to 3 to remove genes expressed in fewer than 3 cells. The cell data is then further filtered, including removing cells with less than 200 or more than 5000 genes, and cells with more than 20% of mitochondrial genes or 5% of hemoglobin genes. No further filtering of genes was performed in this step.

To eliminate the effect of doublet cells, the doubletFinder_v3 function from the DoubletFinder package was used for doublet filtering. The main parameters were set to PCs = 1:20 and pN = 0.25, i.e., based on 20 principal components (PCA), the simulated probability of each cell being labeled as two-cell was 0.25. The filtered data was normalized to the raw counts using the LogNormalize method, which normalizes the total gene expression per cell to 10,000. Next, 2000 highly variable genes were extracted by the FindVariableFeatures function and normalized using the ScaleData function to reduce the effect of technical noise. Subsequently, the data were downscaled by RunPCA and the top 20 principal components were selected for subsequent processing.

For correction of batch effects for multi-sample data, integration was performed using the RunHarmony function from the Harmony package. Using samples as grouping variables (group.by.vars = “sample”), we set the integration strength parameter lambda = 1 and the clustering penalty parameter theta = 2. The Harmony method is based on PCA, which removes systematic biases specific to the dataset through embedding and iterative algorithms to realize batch effect correction, and ultimately the cells of different samples can be well aggregated after integration. After the integration, the cells from different samples can be well aggregated. The ‘umap-learn’ algorithm in RunUMAP was used to perform umap dimensionality reduction of the data to facilitate subsequent visualization. After the batch effect correction was completed, the FindNeighbors function was used to calculate the distance between cells and construct the shared nearest neighbor (SNN) graph; subsequently, the cells were clustered based on Louvain’s algorithm by the FindClusters function, and the resolution parameter was set to 0.6 in order to identify the subpopulations of cells.

For cell annotation, we used the cellmark2.0 database to annotate cell subgroups based on common cell mark genes.

### 2.4 Functional enrichment analysis

We identified differentially expressed genes (DEGs) (|logFC|>0.25; P<0.05) for each cluster using the FindMarkers function in Seurat. Only up-regulated DEGs were obtained for further analysis. GeneID conversion of up-regulated DEGs between populations was performed using the ClusterProfiler function package (v 4.8.2) in R, and enrichment analysis was performed (21). The enrichment analysis included Gene Ontology (GO) and Kyoto Encyclopedia of Genes and Genomes (KEGG) analysis.

### 2.5 Recognizing malignant and non-malignant epithelial cells

The initial copy number variation (CNV) of each cell was estimated using the Infercnvpy package (v0.4.4; https://github.com/icbi-lab/infercnvpy) to distinguish malignant epithelial cells (mECs) from non-malignant epithelial cells (ECs) (22). The algorithm was executed with T cells serving as the normal reference and using default parameters. Subsequently, CNV scores for each cell were calculated using the infercnvpy.tl.cnv_score function (**Supplementary Table S1**).

### 2.6 Identification of prognosis-associated cell subtypes

We employed a computational tool for identifying phenotype-associated cell subpopulations (Scissor) algorithm (v2.0.0) to correlate bulk RNA-seq survival data from TCGA-ESCC with single-cell transcriptomic profiles (23). Patient survival status in TCGA was determined based on clinical follow-up data, where deceased patients (OS event = death) were classified as “worse prognosis”, while surviving patients (OS event = alive) were designated as “good prognosis”. he Scissor analysis was performed specifically on epithelial cells using the following parameters: alpha=0.003, family= “cox”. Scissor^+^ cells were associated with worse prognosis, while Scissor^-^ cells were associated with good prognosis.

### 2.7 Transcription factor analysis

We utilized the pySCENIC Python package (v0.12.1) algorithm to calculate regulon activity scores for malignant epithelial cells (24). First, co-expression modules between transcription factors (TFs) and candidate target genes were inferred using GRNBoost2. Then, genes in each co-expression module were analyzed using RcisTarget to identify enriched motifs (a transcription factor and its potential direct target gene were defined as a regulator). Finally, the activity of each regulator in each cell was assessed using AUCell.

### 2.8 Trajectory analysis

Monocle (v2.28.0) was used to construct pseudotemporal trajectories based on gene expression profiles of malignant epithelial cells (25). After downscaling and cell sorting, all malignant epithelial cells were projected and sorted into trajectories with different branches, and cells within the same branch were considered to have the same cellular state. Branched Expression Analysis Modeling (BEAM) was further performed to identify genes exhibiting branch-dependent expression patterns, where cell fate 1 corresponds to state 2 and cell fate 2 corresponds to state 1.

### 2.9 Analysis of cellular interactions

We utilized the CellChat (v 1.6.1) algorithm to investigate potential interactions between different cell types (26). Among them, a merged Seurat object containing epithelial cells and other cell types in the tumor microenvironment (TME) was used as input to the algorithm. After creating the CellChat objects, we built a reference database using the secretory signaling pathways. Specific receptor-ligand interactions and communication probabilities between different cell types were inferred using the computeCommunProb and computeCommunProbPathway functions, respectively.

### 2.10 Univariate Cox regression survival analysis

The effect of apoptosis-related genes (**Supplementary Table S2**) on patients’ survival risk was assessed using univariate Cox regression survival analysis. We performed multiple testing correction by applying the false discovery rate (FDR) adjustment (Benjamini-Hochberg method) to the univariate Cox regression results. p adj<0.05, HR≠1 was the threshold to screen for genes associated with prognosis. “surv_cutpoint” was obtained to bestcutoff threshold to divide the samples into CTSC high expression group and CTSC low expression group. The survival curves were fitted using the Kaplan-Meier method in the R survival package and visualized using the ggsurvplot function in the survminer package.

### 2.11 Tissue specimen collection

Tumor samples and adjacent normal tissues were surgically resected from 12 patients diagnosed with ESCC at The First Affiliated Hospital Zhengzhou University. The study protocol was reviewed and approved by the Committees for Ethical Review of Research at Zhengzhou University (2022-KY-0149). Written informed consent was obtained from all patients in accordance with the requirements of the Declaration of Helsinki.

### 2.12 Immunohistochemistry

Human tumor or adjacent normal tissues specimens were fixed in 4% formaldehyde, embedded in paraffin, and sliced into 5 µm thick sections using a microtome. After deparaffinization with xylene, the tissue sections were hydrated using ethanol solutions of varying concentrations for antigen retrieval. Subsequently, the sections were treated with a citric acid repair solution (Fuzhou Maixin Biotechnology Development Co., Ltd., China). Next, an appropriate amount of endogenous peroxidase blocker (Beijing Zhongshan Jinqiao Biotechnology Co., Ltd., China) was added, and the sections were incubated for 10 minutes at room temperature. Following this, the sections were washed three times with PBS solution, with each wash lasting 3 minutes. The sections were then blocked with 10% goat serum and incubated with anti-CTSC antibody (1:100; #sc-74590, Santa Cruz, USA) at 4°C overnight. After washing three times with PBS solution, the sections were incubated with a secondary antibody for 30 minutes at 25°C, followed by color development using DAB for 10 minutes. Finally, the sections were counterstained with hematoxylin for 2 minutes to visualize the nuclei and were observed under a microscope (Zeiss, Germany).

### 2.13 Cell culture

ESCC cell line KYSE520 was purchased from American Type Culture Collection (ATCC, USA). Subsequently, cells were cultured in 1640 medium supplemented with penicillin/streptomycin (100 mg/mL) and 10% fetal bovine serum (FBS; Gibco; USA). These cells were incubated at 37°C in 5% CO_2_.

### 2.14 Cell transfection

We performed experiments when KYSE520 cells were grown logarithmically. For transfection, we followed the manufacturer’s instructions and transfected Lipofectamine 3000 Transfection Reagent (Invitrogen, USA) with 5 nmol of siRNA fragments (si-CTSC-1 and si-CTSC-2) targeting CTSC and a negative control (si-NC) (GenePharma, China), into approximately 4×10^5^ KYSE520 cells. The transfection efficiency was subsequently assessed by quantitative reverse transcription-polymerase chain reaction (qRT-PCR). The relevant sequences are listed in **Supplementary Table S3**.

### 2.15 Quantitative real-time PCR

Total RNA was isolated from KYSE520 cells using Trizol reagent (Thermo Fisher Scientific, USA) following the manufacturer’s instructions. Subsequently, reverse transcription was performed using the PrimeScript™ RT kit (Takara, Japan). Next, qRT-PCR was performed using HiScript II Q RT SuperMix for qPCR (TRANS, AU341). GAPDH was used as an internal reference gene for normalization. Relative gene expression was quantitated using the 2^- (△Ct sample - △Ct control)^ method. The sequences of primers used in this study are shown in **Supplementary Table S4**.

### 2.16 Cell counting kit-8

After KYSE520 cells were cultured to good condition, they were incubated under optimal conditions and laid in 96-well plates for CCK8 kit (#KGA317, KeyGEN Bio, China) to detect cell proliferation. siRNA transfection was carried out when the cells were grown to 70% density, and CCK8 experiments were performed 48 hours after transfection. 10 µL of CCK8 reagent was added to each well and incubated for 2 h. After incubation, the 96-well plate was transferred to an enzyme labeling instrument for detection. The absorbance value (OD value) of each well was measured at 450 nm and used to assess cell viability.

### 2.17 5-ethynyl-2’deoxyuridine detection assay to assess cell proliferation

A 10 mM EdU solution (Elab Fluor^®^ 647, elabscience, China) was diluted into cell culture medium to prepare a 2× EdU working solution (20 µM), which was then added to a 6-well plate to achieve a final concentration of 1×. After incubating the cells for 18 hours, they were digested and fixed with fixation/permeabilization buffer for 15 minutes. Subsequently, the cells were washed with washing buffer, followed by the addition of Click reaction solution containing Click Reaction Buffer I, CuSO^4^, Click Additive Solution, and Elab Fluor^®^ 647. The cells were incubated at room temperature in the dark for 30 minutes. Finally, the cells were resuspended in PBS and analyzed using a flow cytometer (NovoCyte 2060R, ACEA Biosciences, China).

### 2.18 Transwell assays and wound healing assays

We further investigated the invasive and migratory capabilities of cells through Transwell assays and wound healing assays, respectively. For the Transwell assay, cells were cultured in serum-free medium for 24 hours. Subsequently, the cells were resuspended in serum-free medium and adjusted to a density of 1×10^4^ cells/mL. A total of 500 µL of medium containing 15% fetal bovine serum (FBS) was added to each well of a 24-well plate, followed by the addition of 200 µL of cell suspension into the Transwell insert. The insert was then placed into the 24-well plate containing complete medium. The cells were incubated in a cell culture incubator for 48 hours. After incubation, the cells in the insert were removed, and any remaining cells were gently wiped off using a PBS-moistened cotton swab. The cells were fixed with 10% methanol for 30 seconds and stained with 0.1% crystal violet for 20 minutes, followed by rinsing with tap water until the background was clear. Finally, 3–5 random fields of view were selected under an optical microscope to count the number of cells that had migrated through the membrane, and images were captured and quantified using Image J software.

For scratch experiments, cells were inoculated in 6-well plates for culture. When the cell confluence reached 90%, a vertical scratch was made in the cell monolayer with a 200 µL pipette tip. After washing 3 times with PBS to remove the scratched cells, serum-free medium was added. Cells were continued to be cultured for 24 h in an incubator at 37°C, 5% CO_2_. Images were taken and recorded at 0 h and 24 h, respectively.

### 2.19 Apoptosis rate was measured by flow cytometry

Cells were transfected with siRNA and cultured for 48 hours. Subsequently, Annexin V-FITC/PI reagent (#C1062, Beyotime, China) was used for cell staining. The results were detected by flow cytometry and analyzed using FlowJo software.

### 2.20 Western blotting analysis

We examined protein expression in ESCC cells using WB. Specifically, cells were lysed using cell lysate and protein concentrations were measured using the BCA protein assay kit (#BL521A, Biosharp, China). The proteins were then separated using a 10% SDS polyacrylamide gel and transferred to a PVDF membrane, which was subsequently soaked in 5% skimmed milk for 2 hours at room temperature, followed by the use of β-actin (1:50,00; #20536-1-AP, Proteintech, China), Caspase-3 (1:800; #24232, CST, China), cleaved-caspase-3 (1:3000; #68773-1, proteintech, China) to the membrane for 1 hour. Subsequently, the membrane was washed three times and then incubated with an HRP-conjugated secondary antibody at room temperature for one hour. The target protein bands were detected using Western blotting detection system (Tanon, China).

### 2.21 Animal experiments

Experiments have been conducted according to the ethical standards, the Declaration of Helsinki, and national and international guidelines. All animal research was conducted according to the ARRIVE (Animal Research: Reporting of *In Vivo* Experiments) guidelines and the AVMA (the American Veterinary Medical Association) guidelines on euthanasia and was approved by the Committees for Animal Ethical Review of Research at Zhengzhou University.

### 2.22 Tumor formation assay

Female BALB/c nude mice of 4–5 weeks old (Shanghai Model Organisms Center, Inc., China) were maintained at SPF conditions, and given a subcutaneous injection of 5 × 106 cells (groups: sh-NC, sh-CTSC) into the right flank, respectively (5 mice/group). Then the subcutaneous xenografts were observed every two days. Tumor volume was measured using calipers and calculated according to the formula: V = length × width2/2. After 28 days, the mice were euthanized and the formed tumors were isolated and weighed then processed into paraffin-embedded tissue samples for further analyses.

### 2.23 Multicolor immunofluorescence

Paraffin-embedded tissue samples were cut into 5-μm sections. Immunofluorescence staining of all slides was performed according to standard protocols using CTSC (#sc-74590, Santa Cruz, USA) and α-SMA (ab5694, abcam, USA) antibodies. The fluorescence intensity of CTSC and α-SMA was measured using Fiji software.

### 2.24 Statistical analysis

We applied the independent samples Mann-Whitney U test for comparison between two groups of samples for continuous variables. The Kruskal-Wallis test was applied for the comparison of samples between the three groups. The chi-square test was applied to categorical variables for comparison between the two groups. The statistical analysis of this study was executed through R v4.0.5 software. A two-tailed P value less than 0.05 was considered statistically significant.

## 3 Results

### 3.1 Identification of cell populations in ESCC tissues based on scRNA-seq

**Figure 1:**
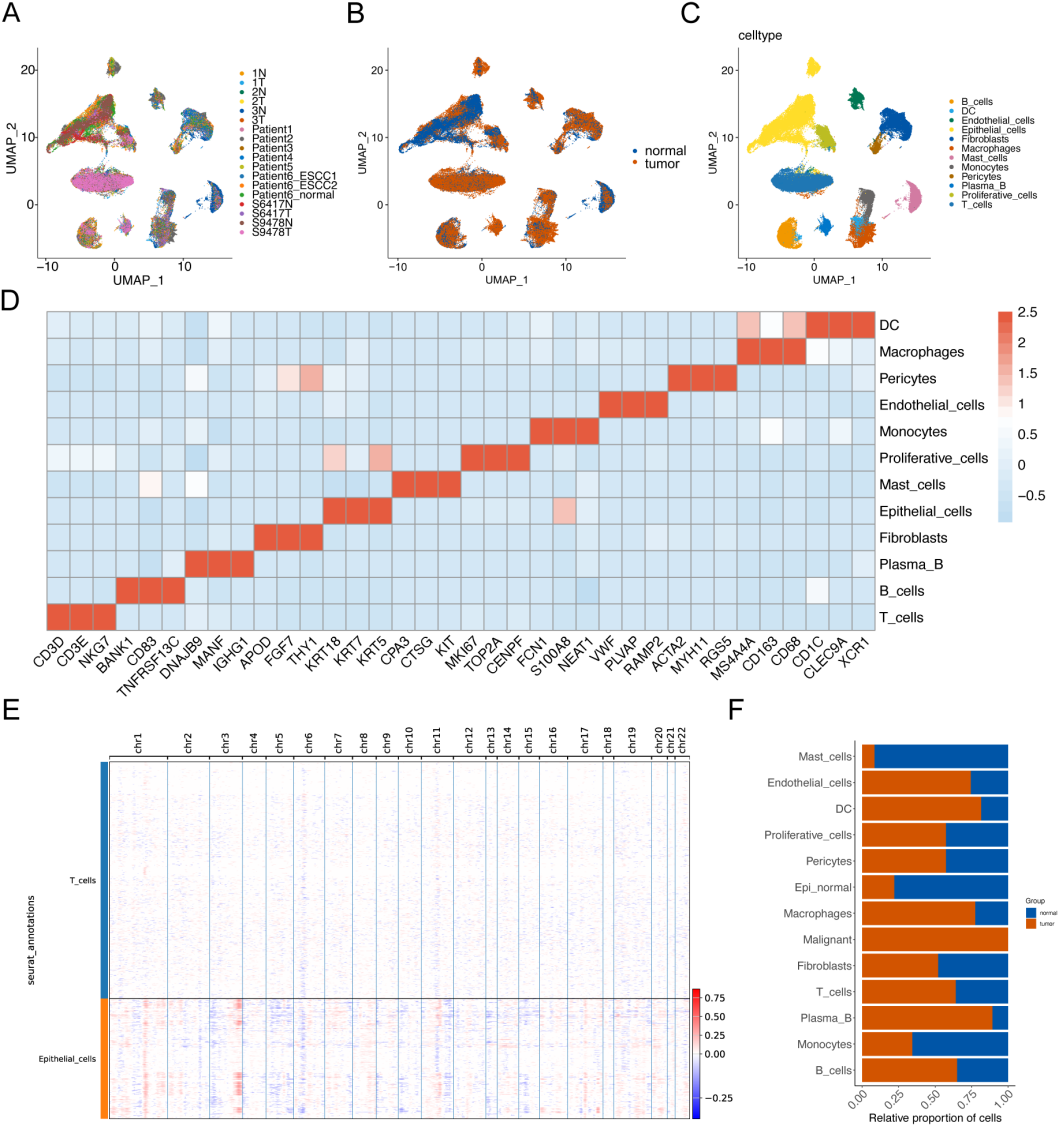
Identification of cell populations in ESCC tissues based on scRNA-seq. UMAP visualization technology performed unsupervised clustering analysis on cells. Each point represents a single cell. **(A)** UMAP visualization is colored according to the sample number. **(B)** UMAP visualization is colored according to the sample grouping. **(C)** UMAP visualization is colored according to the cell type. **(D)** The heat map shows the average expression of typical marker genes of 12 cell types. **(E)** Hierarchical heatmap showing a wide range of copy number variations (CNVs) in epithelial cells (ECs) to identify malignant cells. **(F)** Histogram of cell proportions.

To elucidate the cellular composition within esophageal squamous cell carcinoma (ESCC) tumors, we downloaded scRNA-seq data that included tumor tissue from 12 ESCC tumors and 6 paraneoplastic tissue samples from the Gene Expression Omnibus (GEO) database (GSE196756, GSE188900, GSE221561) (**Figures 1A, B**). After conducting quality control and eliminating batch effects, we obtained a total of 92,714 cells from the primary tumors. Subsequently, based on the expression of cell marker genes, we identified 12 major cell populations: T cells, macrophages, B cells, fibroblasts, epithelial cells, mast cells, proliferative cells, monocytes, endothelial cells, plasma B, DCs and pericytes (**Figures 1C, D**). Subsequently, inferCNV was employed to identify malignant cells, resulting in the identification of a total of 5,355 malignant cells, and there was a large range of copy number variation (CNV) in the epithelial cells (ECs) (**Figure 1E**). This result suggested that the majority of epithelial cells were malignant. Additionally, we compared the composition of cell types in tumor samples with that in normal samples and found that endothelial cells, macrophages, and T cells were more prevalent in the tumor samples (**Figure 1F**).

### 3.2 Integration of phenotype-related bulk data reveals malignant cell subtypes

**Figure 2:**
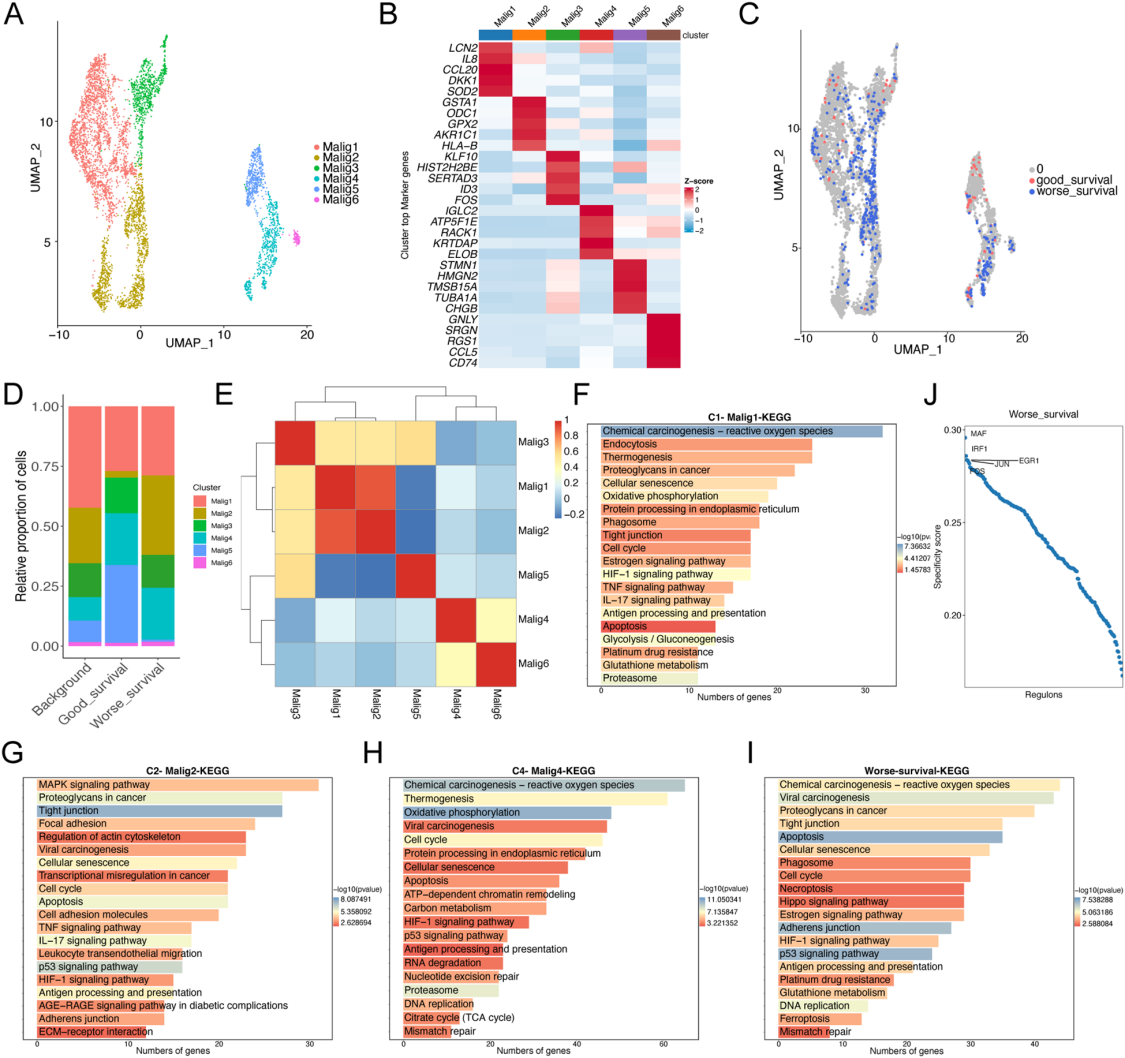
Re-clustering analysis of malignant epithelial cells. **(A)** UMAP plot showing the distribution of 5355 malignant epithelial cells, colored by cluster. **(B)** The heatmap shows the expression of marker genes in malignant cell subtypes. **(C)** UMAP plot showing the distribution of malignant epithelial cells screened by the Scissor algorithm, classified by prognostic risk and protection. Red and blue dots represent cells associated with good and worse prognosis phenotypes, respectively. **(D)** Bar graph showing the proportion of each subtypes of malignant cells in cells associated with prognostic phenotypes. **(E)** Heat map showing the correlation of each subtypes of malignant cells. **(F–H)** Bar graph showing the results of KEGG enrichment analysis of differentially expressed genes in malignant cell subtypes 1 **(F)**, 2 **(G)**, and 4 **(H, I)** Bar graph showing the results of KEGG enrichment analysis of differentially expressed genes in malignant cells with worse prognosis. **(J)** Scatter plot showing regulon specificity score (RSS) in malignant cells with worse prognosis. The top five regulons are highlighted in the figure.

To compare the distribution of malignant cell subclusters in the two samples, we reclustered the malignant cells in the samples to obtain a total of 6 malignant cell subclusters (**Figures 2A, B**). After integrating common transcriptome prognosis-related phenotypic correlation analysis by Scissor, we identified 444 cells as worse prognosis-related subclusters, and the remaining cells were categorized as good prognosis-related subclusters (**Figure 2C**). The malignant cells associated with a worse prognosis were primarily composed of the following subtypes: C1 - Malig1 (128 cells, 28.8% of 444), C2 - Malig2 (147 cells, 33.1%), and C4 - Malig4 (96 cells, 21.6%) (**Figure 2D**). Correlation analysis indicated that Malig1 and Malig2 cells exhibited greater similarity (**Figure 2E**). Furthermore, KEGG enrichment analysis revealed that upregulated genes specific to the Malig1, Malig2, and Malig4 subtypes, as well as worse prognosis-related subclusters, were significantly enriched in the apoptotic pathway (**Figures 2F–I**; **Supplementary Figure S1**). Additionally, we found that worse prognosis-related malignant cells were enriched in the transcription factors, such as IRF1, FOS, EGR1, and JUN, all of which are known regulators of apoptosis (**Figure 2J**).

### 3.3 Dynamics of malignant epithelial cells

**Figure 3:**
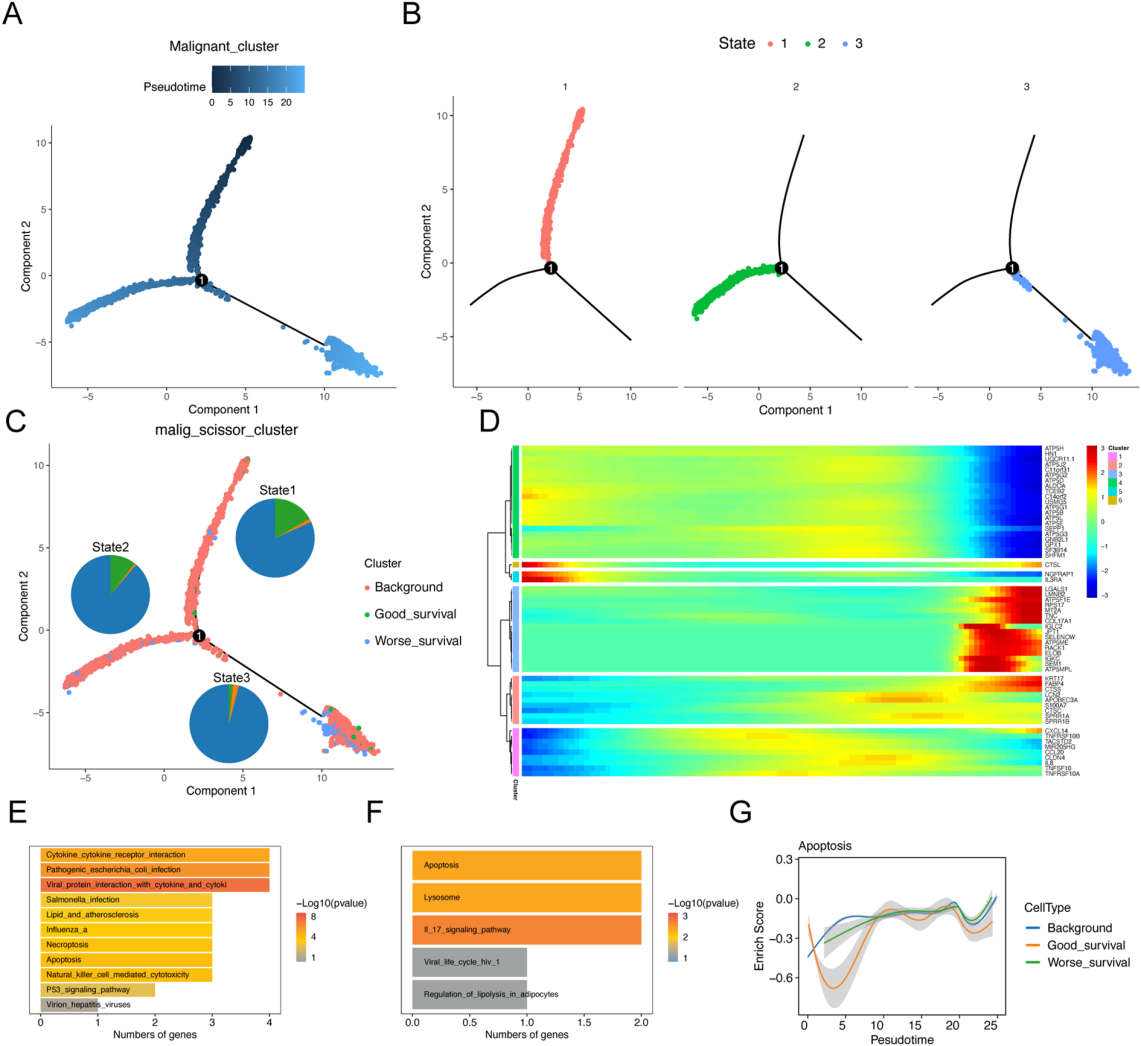
Trajectory analysis of malignant epithelial cell subtypes. Sequential analysis of malignant epithelial cell subtypes by Monocle. **(A)** Colors represent pseudo-time order. **(B)** Cell state trajectory (colors represent different differentiation states). **(C)** The trajectory plot shows the locations of different types of malignant cells, and illustrates the distribution of malignant cells across different trajectory branches using pie charts. **(D)** Heat map showing the dynamic changes of gene expression along pseudo-time. **(E, F)** KEGG enrichment results of cluster1 **(E)** and cluster2 **(F)** genes. **(G)** Two-dimensional graph showing the changes of expression scores of apoptosis-related genes in malignant cell subtypes along pseudo-time.

To understand the dynamics of malignant epithelial cells, we constructed a cellular trajectory to infer differentiation relationships among worse prognosis-related subclusters. Among the six epithelial cell subtypes, we identified three distinct states, with state 1 considered a potential starting point (**Figures 3A, B**). Notably, good survival -related subclusters were predominantly located in state 1, while worse prognosis-related subclusters were primarily found in state 2 and state 3 (**Figure 3C**). Through branching expression analysis modeling (BEAM), we identified 58 branching-dependent genes that may regulate the differentiation process from pre-branching (state 1) to post-branching (state 2 and state 3) (**Figure 3D**). These genes were categorized into six modules (clusters) based on expression similarity, with genes in clusters 1 and 2 significantly enriched in the apoptotic pathway (**Figures 3E, F**). This finding demonstrated that apoptosis played a crucial regulatory role in the differentiation process of malignant epithelial cells. Furthermore, the results of pathway enrichment analysis revealed that the apoptotic pathway exhibited an increase in expression scores along the inferred pseudo-time (**Figure 3G**).

### 3.4 Remodeling of the immune microenvironment in ESCC

**Figure 4:**
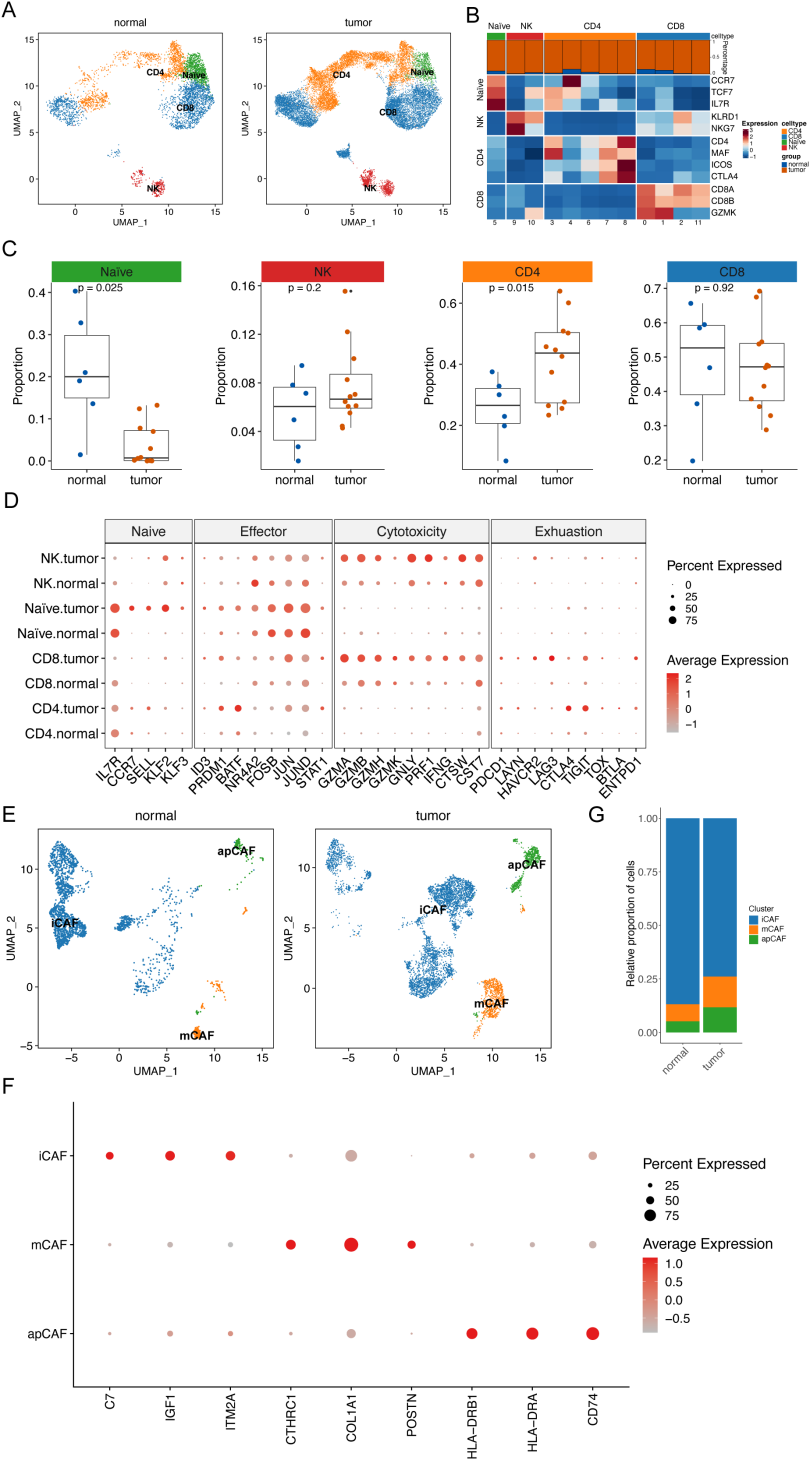
Remodeling of ESCC microenvironment. **(A)** UMAP plot shows the distribution of T cell subtypes in normal tissues and tumor tissues. **(B)** Heat map shows the average expression levels of typical marker genes of T cell subtypes. The bar graph above the heat map shows the relative proportions of T cell clusters in normal tissues and tumor tissues. **(C)** Scatter plot shows the relative proportions of four T cell subtypes in normal tissues and tumor tissues. Wilcoxon rank sum test was applied to determine statistical significance. Each dot represents a sample. **(D)** Bubble plot shows the expression patterns of characteristic genes of four T cell subtypes in normal tissues and tumor tissues. The size of each bubble represents the proportion of T cell subtypes expressing the gene. The color intensity of the bubble represents the normalized value of gene expression level. **(E)** UMAP plot shows the distribution of fibroblast subtypes in normal tissues and tumor tissues. **(F)** Bubble plot shows the expression patterns of characteristic genes of three fibroblast subtypes in normal tissues and tumor tissues. The size of each bubble represents the proportion of T cell subtypes expressing the gene. The color intensity of the bubble represents the normalized value of gene expression level. **(G)** Histogram showing the difference in the proportion of specific cell subtypes in normal tissues and tumor tissues.

The development of ESCC is closely associated with the tumor microenvironment (TME). Single-cell analysis revealed an accumulation of fibroblasts and T cells in the tumor tissues (**Figure 1F**). Consequently, we conducted an unsupervised cluster analysis of T cells. This analysis identified 12 distinct clusters, including one initial T-cell cluster (naïve T cells), two natural killer (NK) cell clusters, five CD4^+^ T-cell clusters, and four CD8^+^ T-cell clusters (**Figures 4A, B**). All T-cell clusters were present in both primary tumor tissues and normal tissues; however, they exhibited heterogeneous cell ratios (**Figures 4B, C**). Notably, NK cells demonstrated enrichment in tumor tissues (**Figure 4C**), while CD8^+^ T cells were enriched in normal tissues (**Figure 4C**). Although the overall cell ratio did not change significantly, CD4^+^ T cells showed upregulation of exhaustion markers in tumor tissues (**Figure 4D**). Additionally, NK cells and CD8^+^ T cells exhibited significant upregulation of cytotoxic markers in tumor samples (**Figure 4D**).

Next, fibroblasts were analyzed through subclustering. We found that these clusters were categorized into three cancer-associated fibroblast (CAF) cell clusters: stromal CAFs (mCAFs), inflammatory CAFs (iCAFs), and antigen-presenting CAFs (apCAFs) (**Figures 4E, F**). Notably, tumor tissues displayed significantly higher ratios of mCAFs and apCAFs, along with lower ratios of iCAFs, compared to normal tissues (**Figure 4G**). This observation suggests a crucial role for CAFs in cancer development.

### 3.5 Cellular communication analysis between ESCC and normal samples

**Figure 5:**
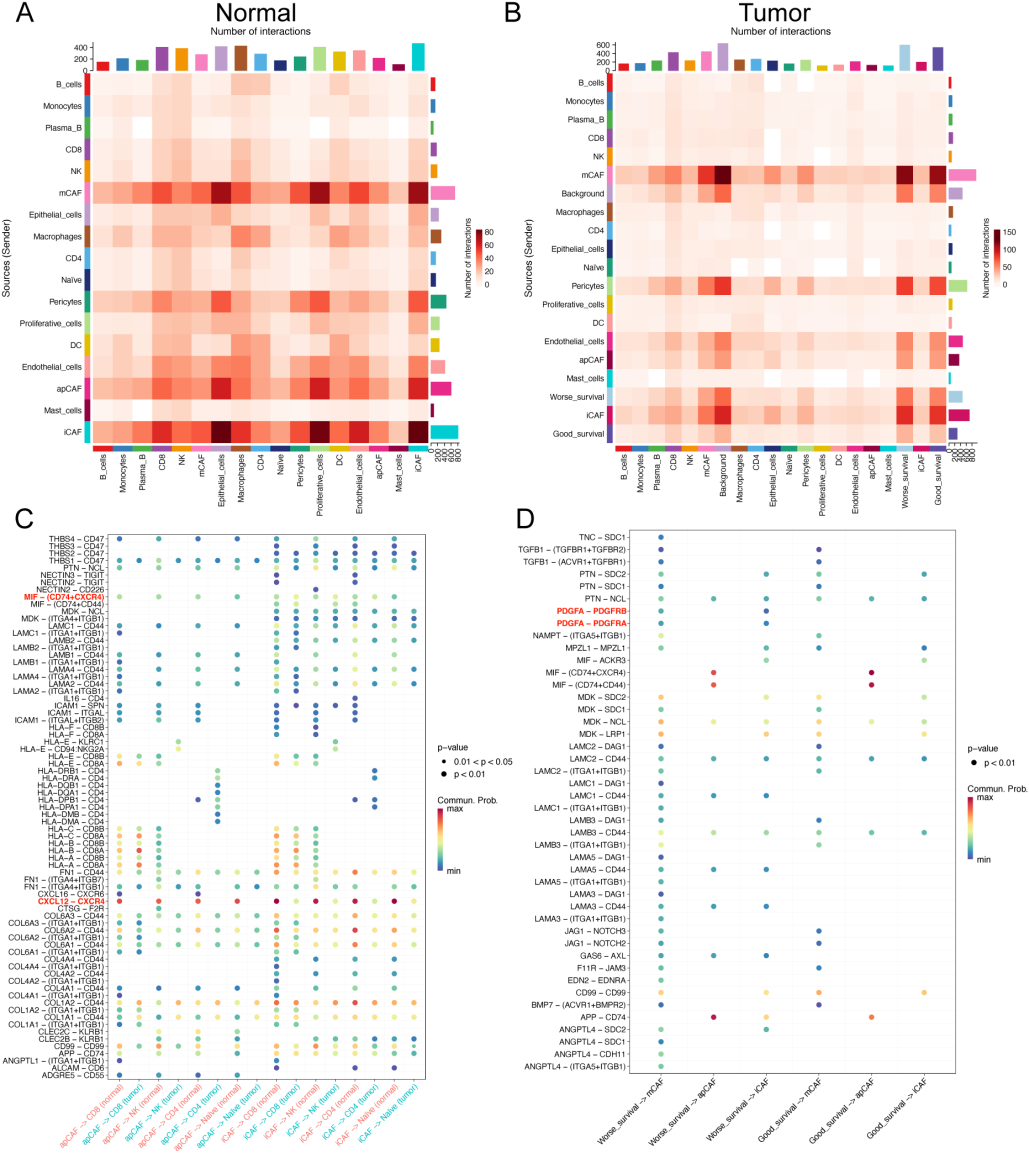
Cellular interactions in the microenvironment of ESCC. **(A, B)** Heat maps show the overall interaction strength between specific cell subtypes in normal **(A)** and tumor **(B)** tissues. **(C)** Bubble plots show the differences in specific ligand-receptor interactions between normal and tumor tissues from apCAFs, iCAFs subtypes to T cell subsets. **(D)** Bubble plots show the differences in specific ligand-receptor interactions between good prognosis and worse prognosis-related cells.

To elucidate the crosstalk among cellular components in the TME, we constructed cellular interaction networks of potential receptor-ligand in normal and tumor tissues (**Figures 5A, B**). Our analysis revealed that communication between different cellular components was significantly more variable in tumor tissue samples compared to normal tissues. In particular, there was an exchange of signals between mCAFs and epithelial cells. In contrast, crosstalk between iCAFs and T cells was diminished in cancer tissue samples.

Furthermore, we found that, compared to tumor tissues, T cell subsets in normal tissues were more regulated by iCAFs and apCAFs through pathways such as CXCL12-CXCR4 and MIF-(CD74^+^CXCR4) (**Figure 5C**). Additionally, worse prognosis-related malignant epithelial cells in tumor samples were also influenced by mCAFs through multiple receptor-ligand pairs, including MDK-SDC1 and MDK-SDC4 (**Supplementary Figure S1**). Notably, mCAFs in tumor samples exhibited specific interactions with worse prognosis-related malignant epithelial cells through MIF-ACKR3, MDK-LRP1, and FGF7-FGFR2 (**Supplementary Figure S2**).

Our analysis revealed enhanced PDGFA-PDGFRA regulatory activity in worse prognosis cells compared to their good prognosis counterparts (**Figure 5D**). Specifically, worse prognosis-related cells exhibited upregulated expression of PDGFA genes (**Supplementary Figure S3A**). Further investigation demonstrated that differentially upregulated genes in tumor-associated mCAFs (matrix-associated cancer-associated fibroblasts) were significantly enriched in the PI3K pathway (**Supplementary Figure S3B, C**), suggesting that CTSC-high cells promote mCAF proliferation through PDGFA ligand-mediated regulation.

### 3.6 Apoptosis-related gene CTSC affected ESCC prognosis and drug resistance

**Figure 6:**
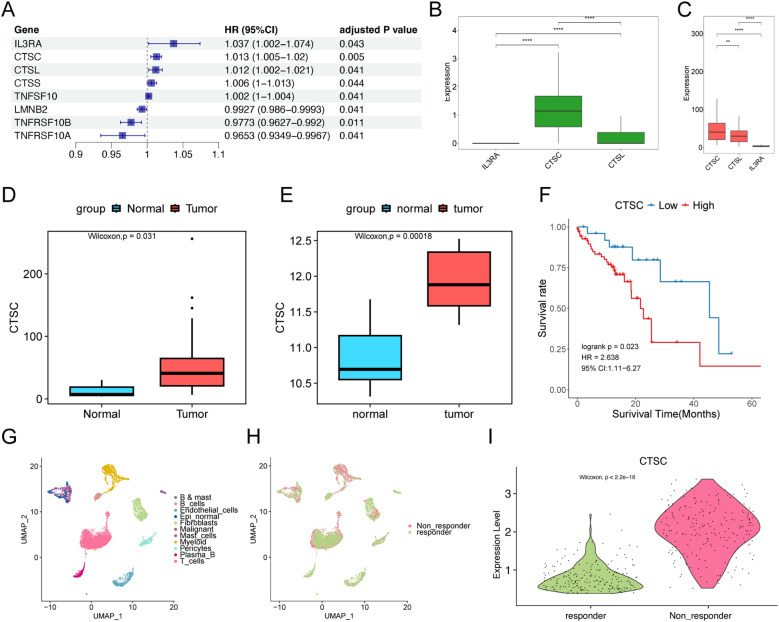
CTSC affects prognosis and drug resistance. **(A)** Univariate Cox regression analysis of apoptosis-related genes. **(B)** Expression of IL3RA, CTSC, and CTSL in epithelial cells associated with worse prognosis in scRNA-seq data. **(C)** Expression of IL3RA, CTSC, and CTSL in TCGA dataset. **(D, E)** CTSC is significantly overexpressed in TCGA **(D)** and GSE75241 **(E)** tumor samples. **(F)** In the TCGA dataset, high-level expression of the CTSC gene indicates a worse prognosis. The P-value of the Log-rank test is less than 0.05 and is considered statistically significant. **(G, H)** A total of 11 types of cells were identified in the GSE203115 dataset, **(H)** involving 2 groups. **(I)** CTSC is significantly overexpressed in malignant cells of non-responder samples. ** p<0.01; **** p<0.0001.

Subsequently, we analyzed apoptosis-related genes and their prognostic correlations using the TCGA-ESCC dataset. We identified a total of eight prognosis-related genes: IL3RA, CTSC, CTSL, CTSS, TNFSF10, LMNB2, TNFRSF10B, and TNFRSF10A. Among these, IL3RA, CTSC, CTSL, CTSS, and TNFSF10 were identified as prognostic risk genes (P<0.05, HR ≠1; **Figure 6A**). We then compared the expression levels of IL3RA, CTSC, and CTSL in malignant cells and found that CTSC exhibited the highest expression in worse prognosis-related malignant cells, as indicated by scRNA-seq data (**Figure 6B**). We downloaded single-cell sequencing data from Ji et al.’s study (DOI: 10.1186/s13073-024-01320-9). The single-cell sequencing samples containing 15 tumors, and 7 paracancerous tissues were analyzed, and a total of 108,699 cells were obtained after data filtering, annotated as 8 types of cells, which were epithelial cells, macrophages, endothelial cells, fibroblasts, plasma cells, T cells, Mural cells, and Mast cells. Subsequently, we analyzed the expression of CTSC in epithelial cells of tumor and normal samples. The results revealed that CTSC was significantly highly expressed in the epithelial cells of tumor samples (**Supplementary Figure S4**, P<0.001). Based on the TCGA database, we observed that CTSC expression was also significantly elevated in ESCC tumor samples (**Figure 6C**). Furthermore, CTSC was markedly overexpressed in tumor samples compared to normal tissues (**Figures 6D, E**). The prognosis for samples with high CTSC expression was significantly worse (**Figure 6F**). Additionally, we found that CTSC expression levels are closely associated with immunotherapy resistance. (**Figures 6G–I**; **Supplementary Figure S5**). These results suggest that high CTSC expression was closely associated with worse prognosis and drug resistance in ESCC, and the role of CTSC in ESCC warranted further investigation.

### 3.7 CTSC was abnormally highly expressed in ESCC tissues

**Figure 7:**
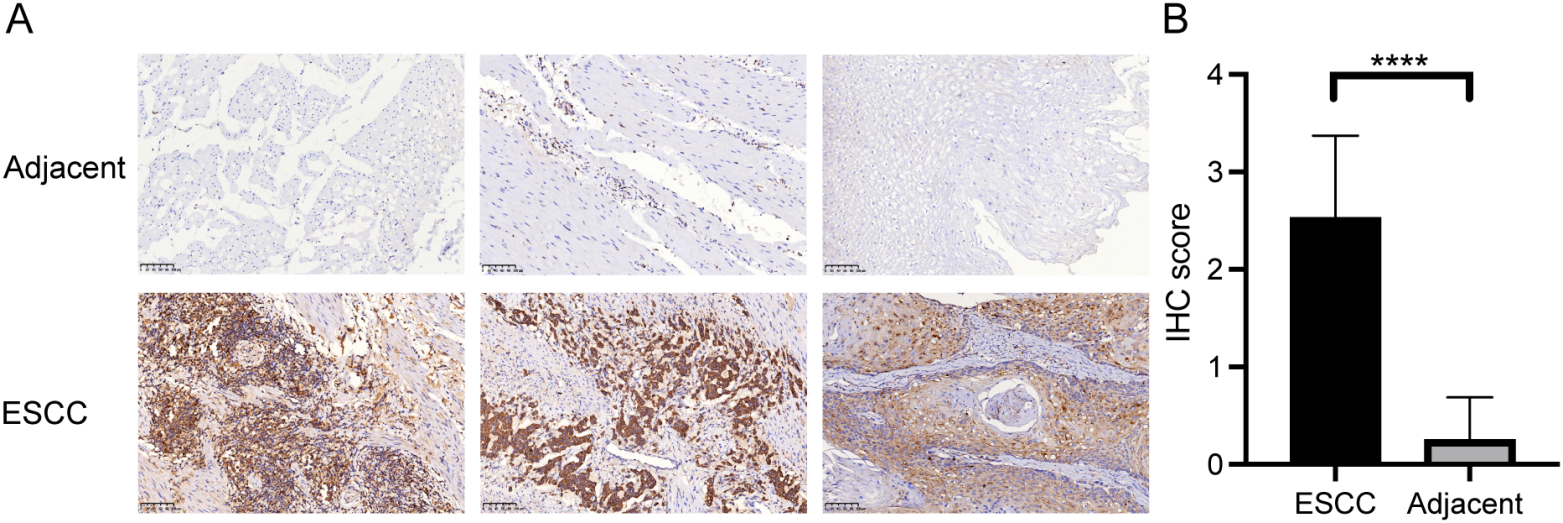
CTSC gene was highly expressed in cancer tissues. **(A)** Representative immunohistochemistry (IHC) staining images of CTSC in adjacent tissues and ESCC tissues. **(B)** Expression of CTSC protein in adjacent tissues and ESCC tissues. Scale bar: 100 μm. ****p < 0.0001.

To validate the expression of CTSC in ESCC using the TCGA database, we enrolled ESCC patients and collected both tumor tissues and adjacent normal tissues. Immunohistochemical analysis revealed that the expression of CTSC was significantly higher in ESCC tissues compared to adjacent normal tissues (P<0.05) (**Figures 7A, B**). This finding was consistent with the predictions from the TCGA database, both indicating elevated levels of CTSC expression in ESCC. Consequently, we hypothesize that CTSC may function as an oncogenic driver, promoting the progression of ESCC.

### 3.8 Knockdown of CTSC suppressed ESCC cell proliferation and migration and promoted apoptosis

**Figure 8:**
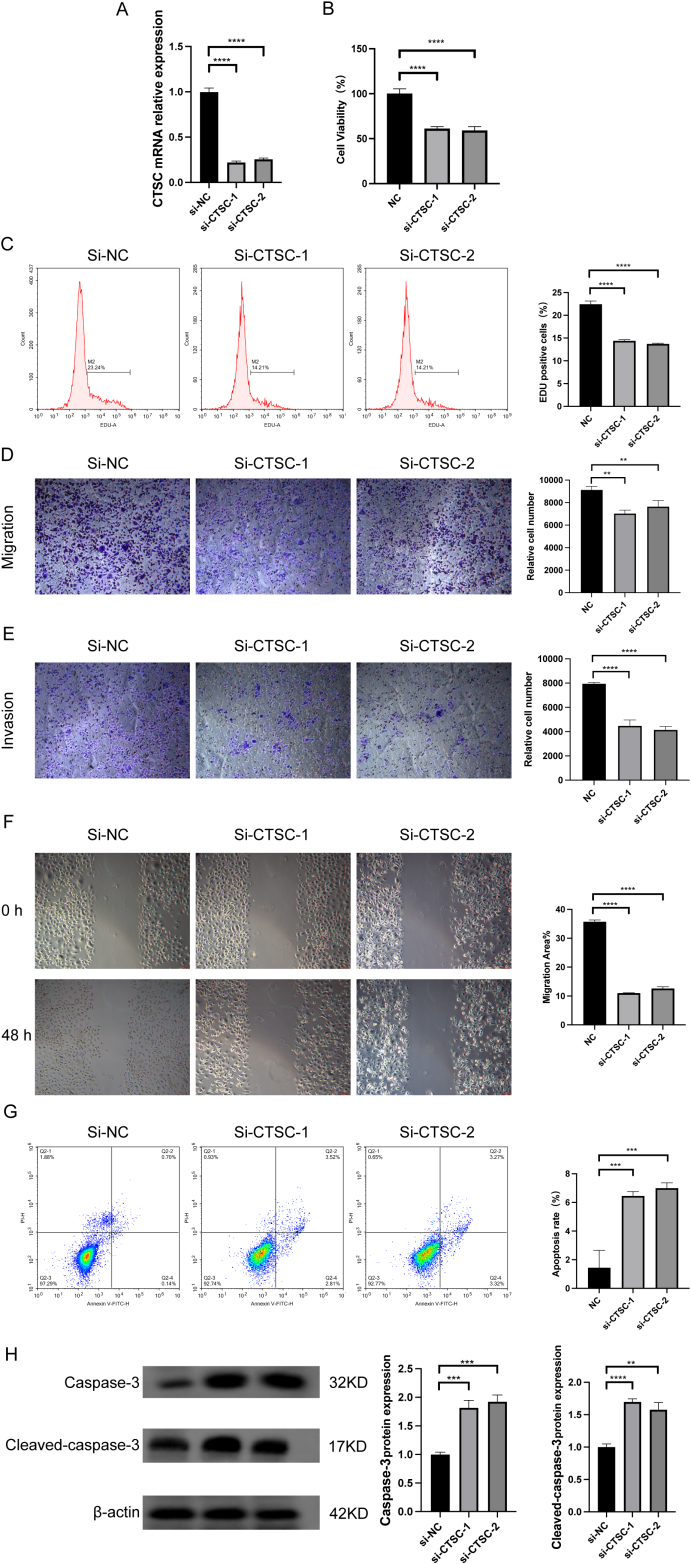
Knockdown of CTSC suppressed ESCC cell proliferation and migration and promoted apoptosis. We designed two siRNAs (si-CTSC-1 and si-CTSC-2) and transfected them into KYSE520 cells to establish CTSC knockdown cell models. **(A)** CTSC mRNA expression was analyzed by RT-qPCR. **(B)** Cell viability was analyzed by CCK8. **(C)** The number of EdU-positive cells was analyzed by flow cytometry. **(D, E)** Cell migration **(D)** and invasion **(E)** ability were evaluated by Transwell. **(F)** Cell migration ability was evaluated by wound healing assays. **(G)** Cell apoptosis was analyzed by flow cytometry. **(H)** caspase 3 and cleaved caspase 3 protein expression was analyzed by WB. **p < 0.01; ***p<0.001; ****p < 0.0001.

To investigate whether CTSC exerts oncogenic effects in KYSE520 cells, we designed two siRNAs (si-CTSC-1 and si-CTSC-2) and transfected them into KYSE520 cells to establish CTSC knockdown cell models. The results demonstrated that transfection with si-CTSC-1 and si-CTSC-2 significantly downregulated the mRNA expression of CTSC, indicating successful silencing of CTSC expression in KYSE520 cells (**Figure 8A**). CCK8 assays revealed that CTSC knockdown markedly inhibited the viability of KYSE520 cells (P<0.05) (**Figure 8B**). Additionally, the number of EdU-positive cells was significantly reduced following CTSC knockdown (P<0.05) (**Figure 8C**). Furthermore, CTSC knockdown suppressed cell migration and invasion (**Figures 8D–F**). Subsequently, flow cytometry was employed to assess cell apoptosis, and the results showed that CTSC knockdown promoted the apoptosis rate (**Figure 8G**). Concurrently, CTSC knockdown significantly increased the expression of apoptosis markers, caspase 3 and cleaved caspase 3 (**Figure 8H**). In summary, these findings demonstrated that CTSC functions as an oncogenic factor, driving cancer cell proliferation and migration while inhibiting apoptosis in ESCC.

### 3.9 Knockdown of CTSC suppressed ESCC progression *in vivo*

**Figure 9:**
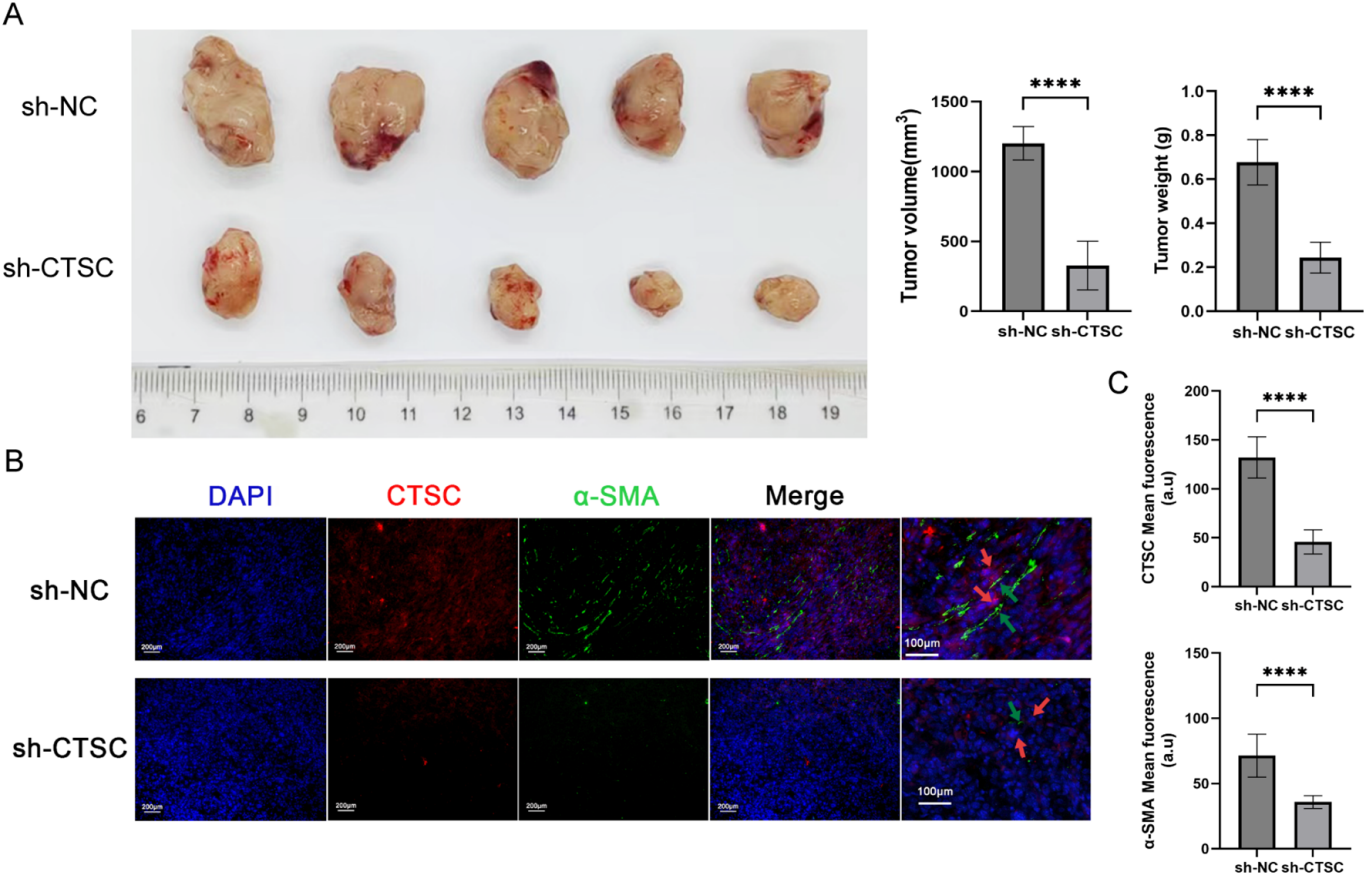
Knockdown of CTSC suppressed ESCC progression *in vivo*. KYSE520 cells transfected with sh-CTSC (n=5) or sh-NC (n=5) were injected subcutaneously into nude mice to construct xenograft tumor models. **(A)** Measurement of tumor volume and tumor weight. **(B)** Multicolor immunofluorescence (mIF) was employed to analyze the spatial location between CTSC+ cancer cells and α-SMA+ fibroblasts. **(C)** The expression of CTSC and α-SMA proteins in tumor tissues. ****p < 0.0001.

To evaluate the impact of CTSC on ESCC progression *in vivo*, we established subcutaneous tumor models in BALB/c nude mice. As shown in **Figure 9A**, the tumor volume and tumor weight of mice in the sh-CTSC group were significantly lower than those in the group with sh-NC (negative control), suggesting that knockdown of CTSC could effectively inhibit tumor growth. In addition, multicolor immunofluorescence (mIF) analysis of tumor tissues showed that the protein levels of CTSC as well as the protein level of fibroblast marker α-SMA were significantly lower in the sh-CTSC group compared with the sh-NC group (**Figures 9B, C**). And the spatial locations of cancer cells and fibroblasts (α-SMA^+^) expressing CTSC were close to each other (**Figure 9B**). Taken together these findings, the results suggest that deprivation of CTSC can inhibit the progression of ESCC.

## 4 Discussion

In this study, we compared the cellular landscape characteristics of primary tumor tissues and adjacent non-cancerous tissues in ESCC. We identified six subtypes of malignant epithelial cells that exhibit mechanisms of deterioration, encompassing distinct transcriptional regulatory networks, coordinated cellular differentiation, and intercellular communication. Additionally, we analyzed the tumor microenvironment (TME) and found that tumor tissues were significantly enriched in immune cells and cancer-associated fibroblasts (CAFs) compared to non-tumor cells. Furthermore, we identified eight apoptosis-related genes associated with prognosis, among which the expression of CTSC was strongly correlated with drug-resistant outcomes in patients undergoing neoadjuvant immunotherapy. Moreover, CTSC was found to promote tumor progression in ESCC. Consequently, CTSC plays a crucial role in driving TME remodeling and the progression of drug resistance in ESCC, making it a potential target for clinical therapy.

It is well known that the tumor microenvironment comprises noncancerous cells and components within the tumor, as well as the molecules they produce and release (27). In this study, we identified 92,714 single cells through scRNA-seq data, which can be categorized into 11 cell clusters. Among these, endothelial cells, macrophages, and T cells are more prevalent in tumor samples. Endothelial cells play a crucial role in tumor angiogenesis. Compared to normal tissues, endothelial cells in tumor tissues undergo metabolic remodeling, including abnormal glucose metabolism, amino acid metabolism, and fatty acid metabolism (28). These metabolic abnormalities not only meet the energy demands of endothelial cells for their proliferation and migration but also provide the necessary metabolic substrates and regulatory signals for tumor angiogenesis (29, 30). In ESCC, an enrichment of specific endothelial cell subtypes has been observed (31). ESCC cells can stimulate the angiogenesis of endothelial cells, thereby promoting tumor migration (32). Macrophages are a crucial component of the mononuclear phagocyte system and play significant roles in immune system regulation and angiogenesis (33). Additionally, macrophages are closely associated with tumors. Under the influence of various cytokines, macrophages can polarize into two distinct functional forms: pro-inflammatory, tumor-suppressive M1 macrophages and anti-inflammatory, tumor-supportive M2 macrophages (33). Macrophages that infiltrate the tumor microenvironment (TME) are referred to as tumor-associated macrophages (TAMs). TAMs are typically M2-like anti-inflammatory immune cells that are linked to malignant disease progression, drug resistance, and worse prognosis. For instance, CCL22 secreted by TAMs contributed to cisplatin resistance in ESCC cells (34). The infiltration of M2 macrophages promoted the progression of ECC (35). Previous studies have also found that T cells were present in large numbers in ESCC tissues (36). Additionally, T cell infiltration in human cancers should be considered as a true regulator of cancer growth (37)). Combined with the results of the present study, these insights emphasize that endothelial, macrophage, and T-cell cells play important roles in TME and drive ESCC progression. In this study, we observed that CD4+ T cells in tumor tissues exhibited upregulation of exhaustion-related molecular markers, while CD8+ T cells were predominantly expressed in adjacent normal tissues. These findings suggest that the infiltration of CD4+ T cells is associated with carcinogenesis, whereas the absence of CD8+ T cells is linked to tumor immune evasion.

Malignant epithelial cells were also identified in this study, and according to the Scissor algorithm, relevant cell populations with worse prognosis were identified. Worse prognosis-related malignant cells were enriched in apoptosis-related transcription factors, such as IRF1, FOS, EGR1, and JUN. Among these, IRF1 exerts its tumor-suppressive effects by promoting ferroptosis and apoptosis, thereby inducing tumor cell death (38). In contrast, FOS, EGR1, and JUN inhibit apoptosis, exacerbating cancer progression (39–41). In addition, this study also found that the apoptotic pathway exhibited elevated expression scores along the extrapolated pseudo-times, which also suggests that apoptosis played a key regulatory role in malignant epithelial cell differentiation.

Subsequently, this study analyzed the correlation between apoptosis-related genes and prognosis using TCGA-ESCC data. Through univariate Cox regression analysis, eight prognosis-related genes were identified: IL3RA, CTSC, CTSL, CTSS, TNFSF10, LMNB2, TNFRSF10B, and TNFRSF10A. Among these, IL3RA, CTSC, CTSL, CTSS, and TNFSF10 were identified as risk genes for worse prognosis (HR>1). Notably, we found that CTSC exhibited the highest expression in malignant cells with worse prognosis in scRNA-seq data, and its expression was also the highest in TCGA tumor samples. Previous studies have also demonstrated that CTSC was aberrantly expressed in various tumors, such as glioma, colorectal cancer, and liver cancer, and was closely associated with worse patient prognosis (42–44). These findings are consistent with the results of the present study, suggesting that CTSC may be a key gene driving worse prognosis in ESCC. In addition, CTSC has been reported to be involved in the process of immune evasion. Dang et al. found that CTSC overexpression promoted the recruitment of myeloid-derived suppressor cells (MDSCs) and tumor-associated macrophages (TAMs) through the CSF1/CSF1R axis, facilitating immune evasion and thereby enhancing cancer cell resistance to immunotherapy (43). Interestingly, this study also observed that CTSC was significantly highly expressed in malignant epithelial cells of patients who did not respond to neoadjuvant immunotherapy. Combined with previous findings, these results suggest that CTSC may promote resistance to immunotherapy in ESCC.

However, the role of CTSC in ESCC remains unclear. Therefore, we investigated the specific functions of CTSC through *in vitro* experiments. The results revealed that, on one hand, knockdown of CTSC inhibited the proliferation and migration of ESCC cells; on the other hand, as an apoptosis-related gene, knockdown of CTSC promoted caspase 3-mediated apoptosis. These findings are consistent with previous studies. Wang et al. demonstrated that overexpression of CTSC significantly enhanced cancer cell viability, proliferation, migration, and invasion, whereas inhibition of CTSC expression suppressed these biological phenotypes (45). In addition, this study also validated the function of CTSC *in vivo*. The results showed that knockdown of CTSC inhibited tumor growth *in vivo* and reduced the protein of the fibroblast marker α-SMA. The above results, suggest that knockdown of CTSC can inhibit the progression of ESCC. These results confirm that CTSC functions as an oncogenic factor, driving tumor progression.

Although this study comprehensively explored the characteristics of the TME in ESCC and identified prognostic risk genes associated with ESCC malignancy, there are still limitations. On one hand, due to the limited availability of non-responsive ESCC specimens following NAT, this study could not clinically validate the association between CTSC expression and NAT treatment efficacy. Future multicenter, multi-platform studies should be conducted to recruit larger cohorts of NAT-resistant ESCC patients, which would enable definitive demonstration of the relationship between CTSC overexpression and NAT resistance. Additionally, the molecular mechanisms by which CTSC drives ESCC progression need to be elucidated in subsequent studies. Additionally, future studies should incorporate rescue experiments (e.g., CTSC overexpression) and pharmacological inhibition (using CTSC-specific inhibitors such as E64) to comprehensively characterize CTSC’s functional role, thereby strengthening the causal evidence and excluding potential off-target effects.

In summary, this study comprehensively compared the cellular landscape characteristics between primary tumor tissues and adjacent non-cancerous tissues in ESCC. Additionally, we identified eight apoptosis-related genes associated with prognosis, among which the expression of CTSC was closely correlated with resistance outcomes in patients receiving neoadjuvant immunotherapy. *In vitro* studies revealed that CTSC promotes tumor progression in ESCC. Therefore, CTSC plays a crucial role in driving TME remodeling and the progression of drug resistance in ESCC, making it a potential target for clinical therapy.

## Data Availability

The datasets presented in this study can be found in online repositories. The names of the repository/repositories and accession number(s) can be found in the article/**Supplementary Material**.

## Supplementary material

Supplementary Figure 1 The apoptosis pathway scores of malignant cell subpopulations.

Supplementary Figure 2 Bubble plots show the differences in specific ligand-receptor interactions between normal and tumor tissues from mCAFs to epithelial cell subtypes.

Supplementary Figure 3
**(A)** Violin plot showing the expression of PDGFA between good prognosis and worse prognosis-related cells. **(B)** Volcano plot was used to display the differentially expressed genes of mCAFs between the two groups. **(C)** Dot plot was used to show that the upregulated genes of mCAFs in the tumor group were significantly enriched in the PI3K pathway.

Supplementary Figure 4
**(A)** UMAP shows the cell types identified in 15 tumors and 7 paracancerous tissues. **(B)**The dot plot displays the expression of marker genes across different cells. **(C)** The violin plot illustrates the expression of CTSC in epithelial cells from tumor and paracancerous tissues.

Supplementary Figure 5 T cells were co-cultured with tumor cells and treated with a combination of drugs. The viability of tumor cells was measured at different time points.
