# Supplementary figures and images for: Identification of CTSC-driven progression in ESCC by single-cell sequencing and experimental validation
Source: Front Immunol. 2025 Jul 16;16:1585139. doi: 10.3389/fimmu.2025.1585139 (PMC12307155; doi:10.3389/fimmu.2025.1585139)

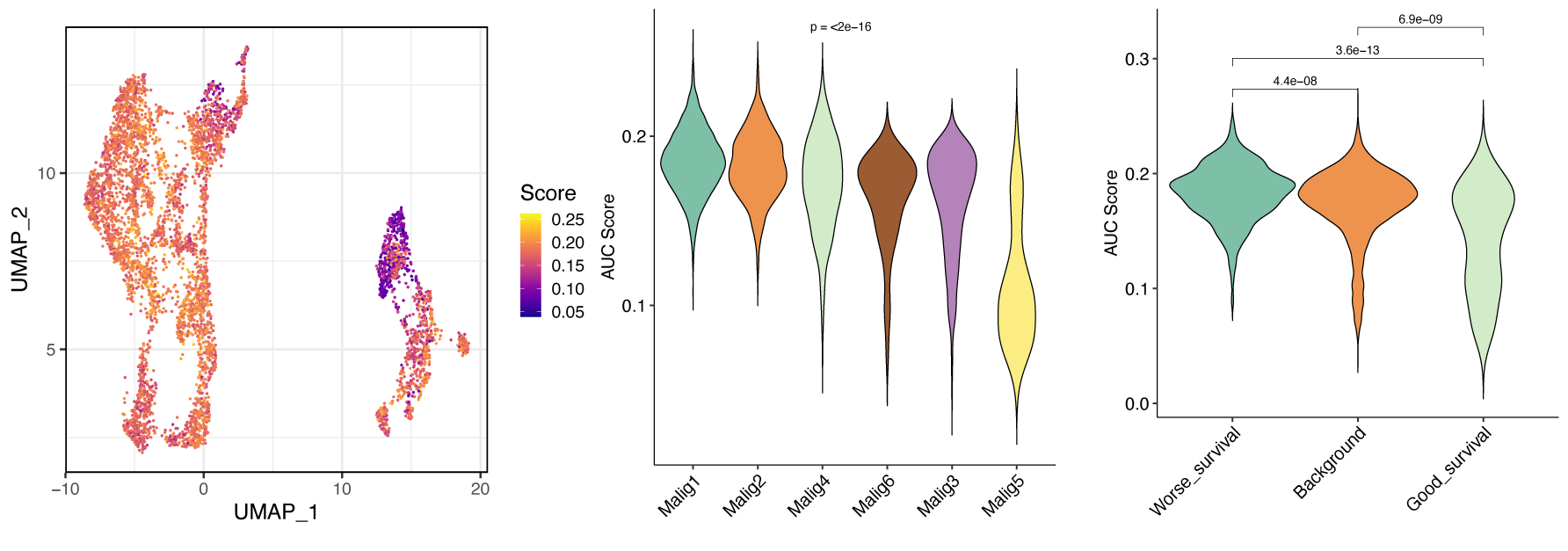

Supplement: Supplementary Figure 1 — The apoptosis pathway scores of malignant cell subpopulations. [file Image1.tif]

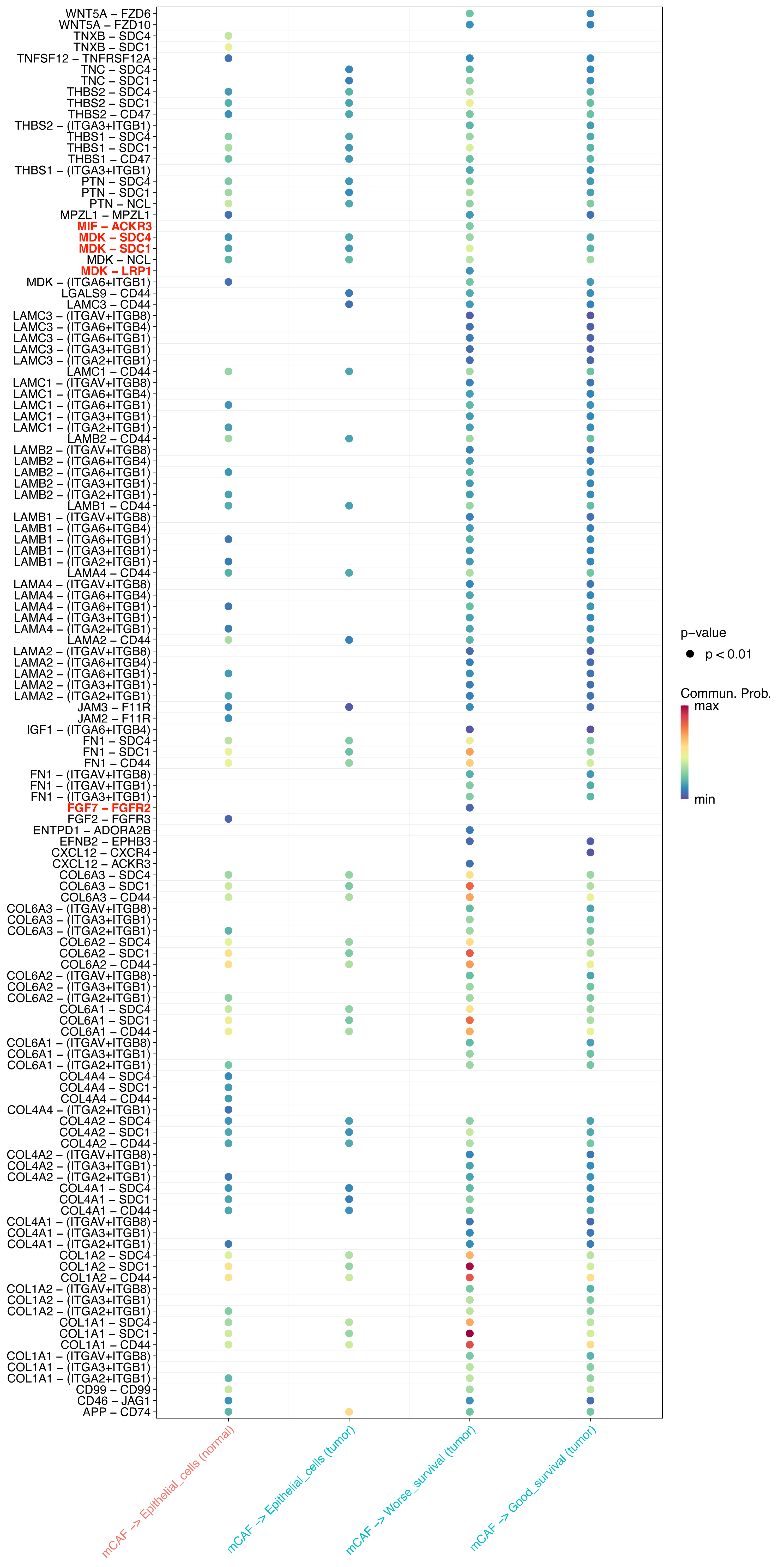

Supplement: Supplementary Figure 2 — Bubble plots show the differences in specific ligand-receptor interactions between normal and tumor tissues from mCAFs to epithelial cell subtypes. [file Image2.tif]

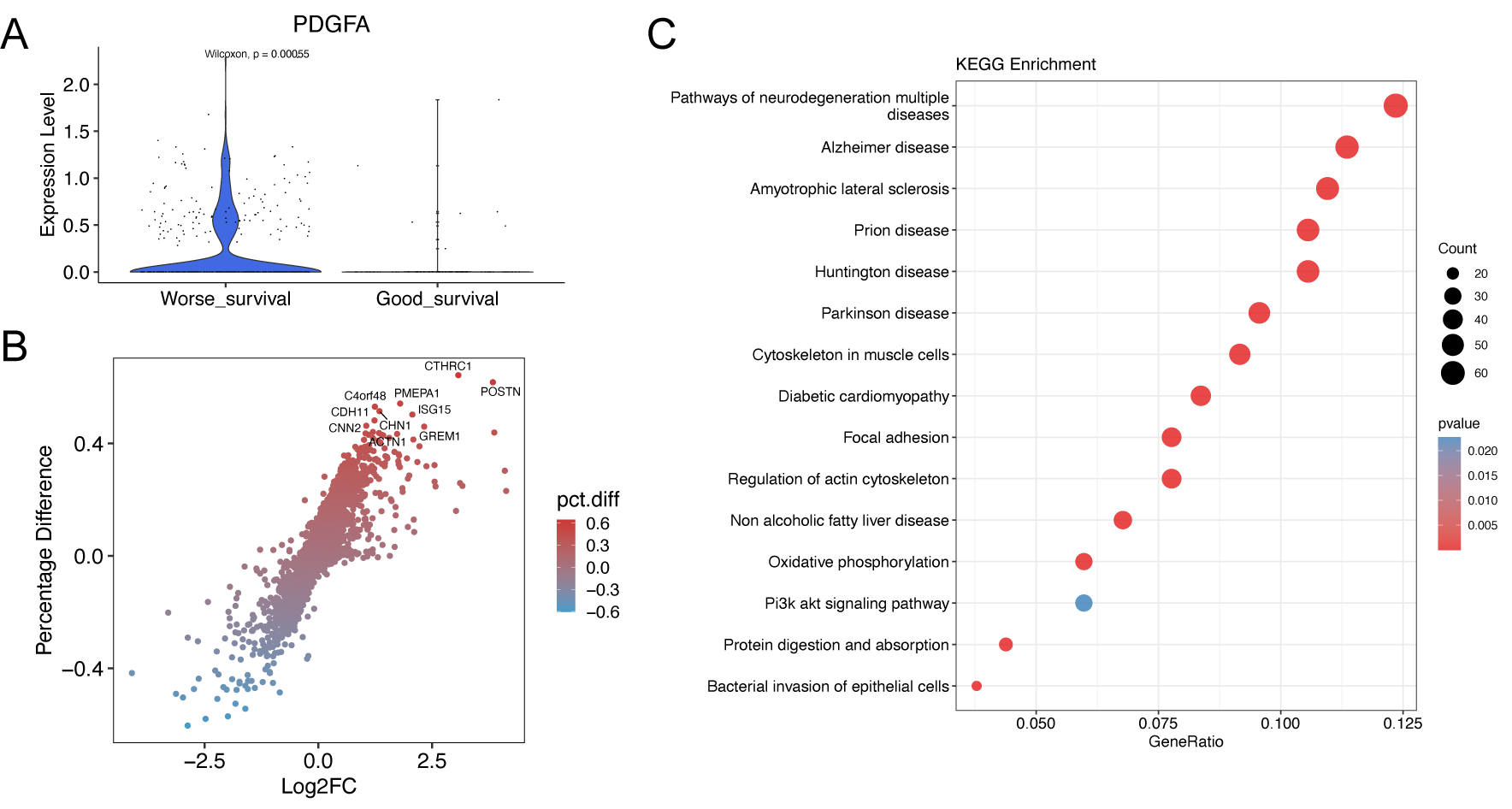

Supplement: Supplementary Figure 3 — (A) Violin plot showing the expression of PDGFA between good prognosis and worse prognosis-related cells. (B) Volcano plot was used to display the differentially expressed genes of mCAFs between the two groups. (C) Dot plot was used to show that the upregulated genes of mCAFs in the tumor group were significantly enriched in the PI3K pathway. [file Image3.tif]

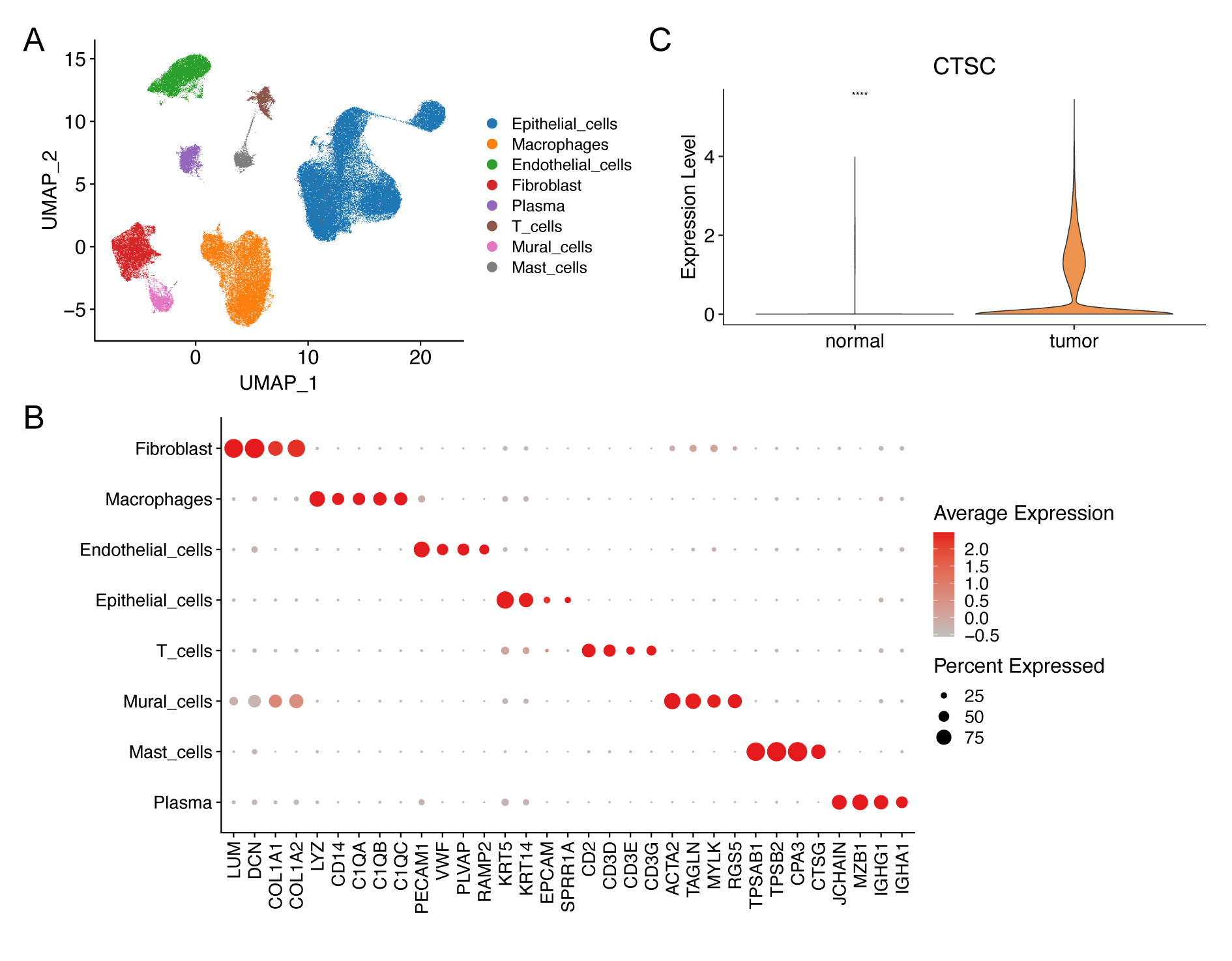

Supplement: Supplementary Figure 4 — (A) UMAP shows the cell types identified in 15 tumors and 7 paracancerous tissues. (B)The dot plot displays the expression of marker genes across different cells. (C) The violin plot illustrates the expression of CTSC in epithelial cells from tumor and paracancerous tissues. [file Image4.tif]

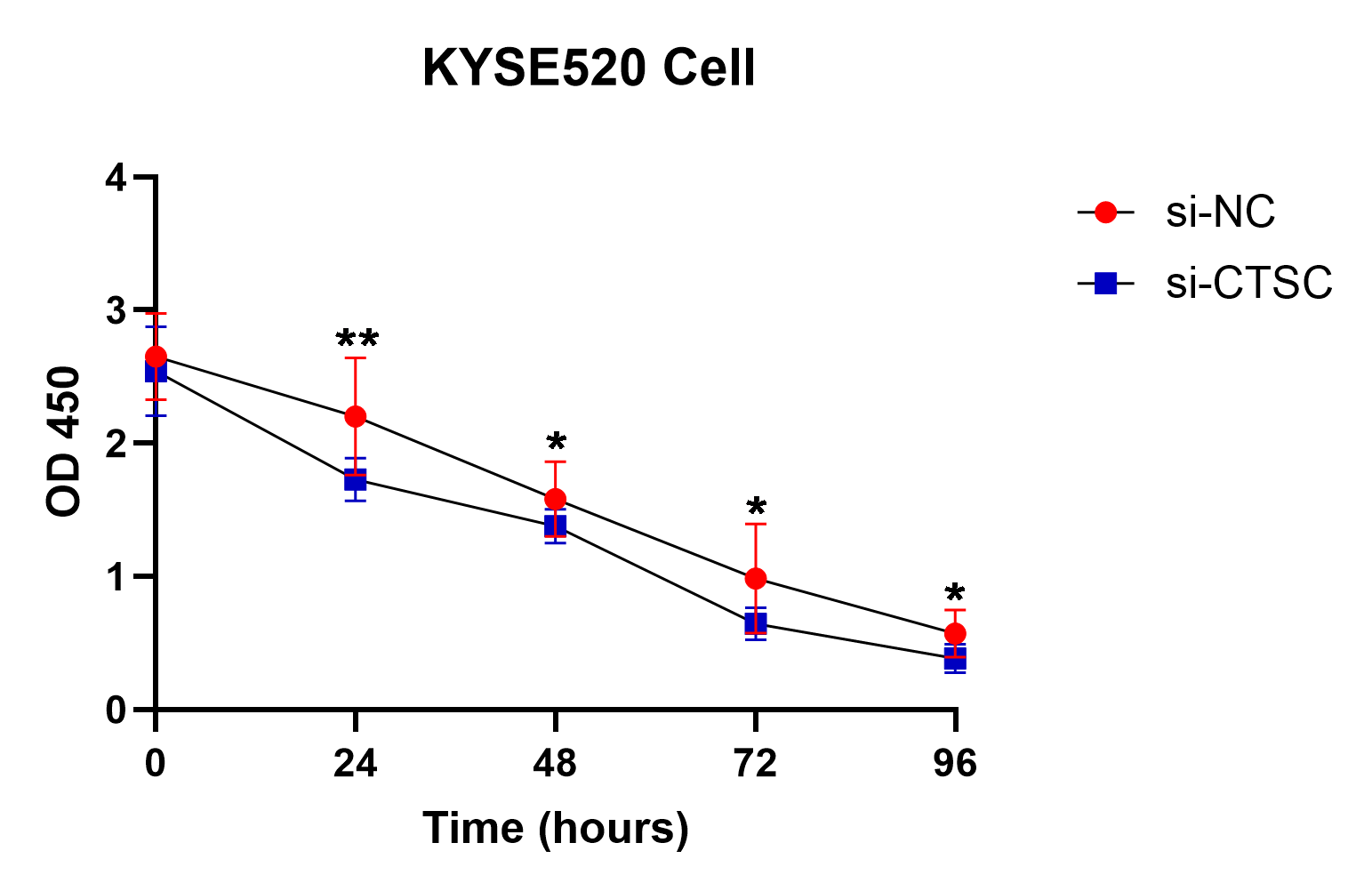

Supplement: Supplementary Figure 5 — T cells were co-cultured with tumor cells and treated with a combination of drugs. The viability of tumor cells was measured at different time points. [file Image5.tif]
